# Supplementary material for: Compositional adaptability in NPM1-SURF6 scaffolding networks enabled by dynamic switching of phase separation mechanisms
Source: Nat Commun. 2018 Nov 29;9:5064. doi: 10.1038/s41467-018-07530-1 (PMC6265330; doi:10.1038/s41467-018-07530-1)
Supplement: Supplementary file 1 — Supplementary Information [file 41467_2018_7530_MOESM1_ESM.pdf]

SUPPLEMENTARY INFORMATION

Compositional adaptability in NPM1-SURF6 scaffolding networks enabled by dynamic switching of phase separation mechanisms

M.C. Ferrolino, *et al.*

SUPPLEMENTARY METHODS

Determination of PEG partitioning within dense and light phases

In order to show that PEG is not incorporated into the scaffold of the homotypic NPM1 droplets, we determined the distribution of PEG molecules between the dense and light phases. To do this we spiked in 10 μM of PEG (MW = 10 kDa) conjugated with TAMRA dye, PEG-TAMRA (Creative PEGWorks, Chapel Hill, NC) into PEG-containing buffers before addition of 10 μM NPM1 to form homotypic droplets to final PEG concentrations of 5%, 15% and 30%. DIC and fluorescence images of the droplets were acquired using a Zeiss LSM 780 NLO point scanning confocal microscope. The partition coefficient of PEG-TAMRA was determined by dividing the mean intensity of the entire droplet area (inside) over the mean intensities of several ROIs outside droplets. Correction factor for TAMRA fluorescence quantum yield at high viscosities in the dense phase was applied. To determine the correction factor, 5 μM free TAMRA dye was added to standard water-glycerol (0%, 20%, 40%, 60%, 80%, 90%, 95% and 100% % glycerol in water) solutions. Solutions were transferred to a 96-well plate. Samples were excited at 525 nm and the emission fluorescence signal in the 530-700 nm range was integrated. The emission range was chosen to match the band pass used in the corresponding confocal microscopy imaging experiments. From this, TAMRA intensities were estimated to increase 1.5-fold in highly viscous solutions. PEG-TAMRA fluorescence inside the droplets were divided by the correction factor, 1.5. Mean of the ratios of intensities were determined for 40 or more droplets per buffer condition.

Fluorescence anisotropy

S6N or ΔN-NPM in 10 mM Tris 150 mM NaCl 2 mM DTT pH 7.5 were serially diluted to final concentrations of 0.01 μM to 10 μM and added to 20 nM ΔN-NPM1-A488 on 384-well microplates (Greiner Bio-One International, Kremsmünster, Austria). Fluorescence anisotropy was measured at 25°C using a CLARIOstar microplate reader (BMG Labtech, Ortenberg, Germany). The dissociation constant, K_d, of S6N for ΔN-NPM1 was determined from the fitted binding isotherm [1].

Covalent crosslinking

Homotypic droplets were prepared by mixing 20 μM NPM1 with 15% Ficoll-70 in 10 mM sodium phosphate, 150 mM NaCl pH 7.0. Ficoll-70 was used as an alternative crowding agent to PEG because it did not cause smearing of protein bands on SDS-PAGE. The viscosity of

15% Ficoll-70 is close to that of 5% PEG [2]. Droplets were treated with 50 μM DSP [dithiobis(succinimidyl propionate)] at different time points after mixing and incubated for two minutes. Crosslinking reactions were quenched with 50 mM Tris pH 7.5. Half of the reaction mix was reduced with 50 mM DTT to break intermolecular crosslinks mediated by DSP. The non-reduced and reduced crosslinked droplet solutions were boiled for 10 min and ran on a denaturing 10% bis-tris SDS-PAGE (Thermo Fisher Scientific, Waltham, MA). The band intensities corresponding to the monomer NPM1 under non-reducing (Inred) and reducing (Ired) conditions were quantified using ImageQuant analysis software (GE Life Sciences, Pittsburgh, PA). The percent crosslinked NPM1 monomers was determined from the equation:

$$\% \, NPM1 \, (crosslinked) \; = \left(1 - \frac{Inred}{Ired}\right) \text{ x } 100$$

The same crosslinking reactions were performed with 20 μM NPM1 in 10 mM sodium phosphate, 150 mM NaCl pH 7.0, in the absence of crowding agent, as a control for crosslinking under one-phase conditions.

Supplementary Table 1. Concentrations of NPM1 in the dense and light phases of homotypic droplets

Buffer	[NPM1] dense, mM*	[NPM1] light, μM**
0% PEG	---	14.1 ± 2.4
5% PEG	4.6 ± 0.2	2.9 ± 0.3
15% PEG	6.5 ± 0.7	0.5 ± 0.1
30% PEG	8.9 ± 1.2	0.1 ± 0.01

*Concentrations of the dense phases were determined for n ≥ 343 droplets per PEG concentration, using fluorescence microscopy.
**Concentrations for the light phases were determined using fluorescence spectroscopy for n ≥ 6 samples.
Values represent mean ± s.d.

Supplementary Table 2. Partitioning of PEG-TAMRA inside and outside homotypic NPM1 droplets formed from different percentages of PEG

Buffer	PEG-TAMRA intensities inside/outside droplet
5% PEG	0.7 ± 0.1
15% PEG	0.9 ± 0.1
30% PEG	0.9 ± 0.1

Ten micromolar PEG-TAMRA (MW = 10 kDa) was added to PEG containing buffers prior to mixing with 10 μM NPM1 to form droplets. Ratios of intensities (inside/outside) were determined for droplets in 5% (n=51), 15% (n=40) and 30% (n=100) PEG buffer. Fluorescence intensities in the dense phase were corrected for effect of viscosity (See Supplementary Methods). Values represent mean ± s.d.

Supplementary Table 3. Mobile fractions NPM1 and S6N inside droplets from different NPM1:S6N ratios determined from FRAP

[S6N]/[NPM1]	Mf (NPM1)	Mf (S6N)
0	0.32 ± 0.03	N/A
0.25	0.17 ± 0.02	0.64 ± 0.05
0.5	0.40 ± 0.04	0.67 ± 0.06
1	0.51 ± 0.08	0.69 ± 0.06
2	0.64 ± 0.05	0.79 ± 0.03
4	0.7 ± 0.06	0.97 ± 0.05

Mf (NPM1) and Mf (S6N) are the average mobile fractions for NPM1 and S6N, respectively, derived from fitted FRAP curves of individual droplets. Values represent mean ± s.d. for n ≥ 10 droplets.

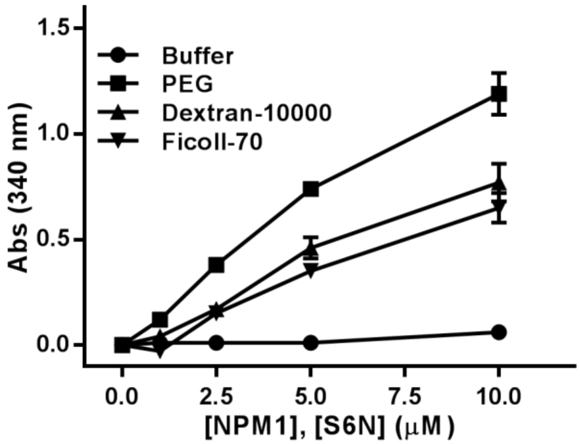

Supplementary Fig. 1. LLPS of NPM1 and S6N in the presence of different types of crowding agents. Turbidity measurements for protein solutions prepared at different NPM1 and S6N concentrations keeping a [S6N]/[NPM1] value of 1.0 with 15% PEG (MW = 8 kDa; squares), Dextran-10000 (MW =10 kDa; triangles) or Ficoll-70 (MW = 70 kDa; inverted triangles) in 10 mM Tris, 150 mM NaCl, 2 mM DTT, pH 7.5 buffer. Turbidity was determined by measuring the absorbance at 340 nm; measurements were made 10 min after mixing. Values represent mean ± s.d. for n = 3.

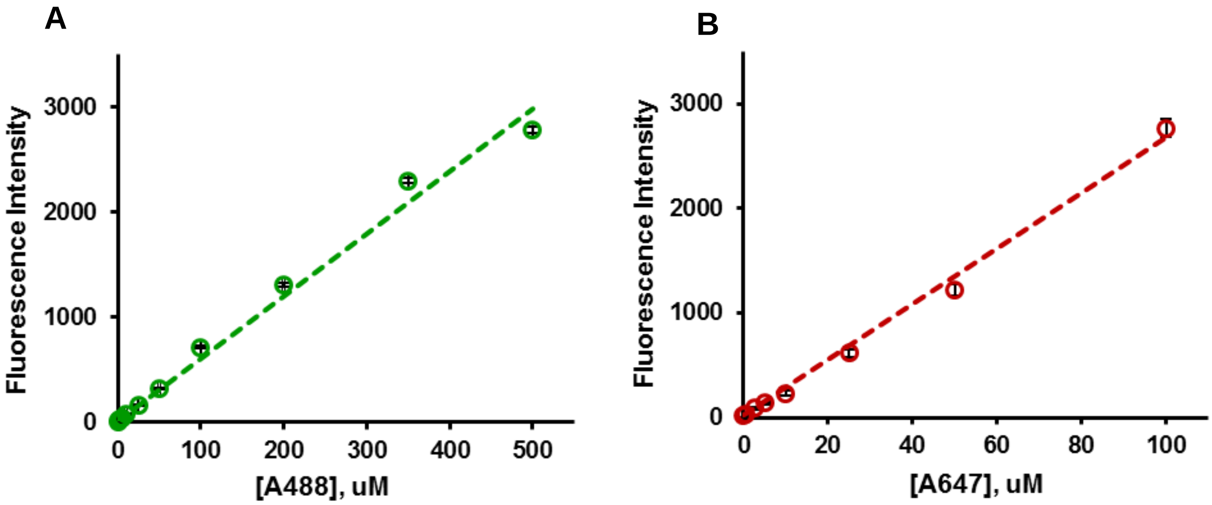

Supplementary Fig.2. Calibration curves generated from free fluorescent dyes. Mean fluorescence intensities were determined from microscopy images of standard solutions of Alexa-488 dye **(A)** and Alexa-647 dye **(B)** in 5% PEG. The fluorescence intensities under these acquisition parameters linearly correlated with the concentrations of Alexa-488 (R^2=0.9897) and Alexa-647 (R^2=0.9958). Similar calibration curves were also constructed in 15% and 30% PEG. Values represent mean fluorescence intensities ± s.d. across the entire image.

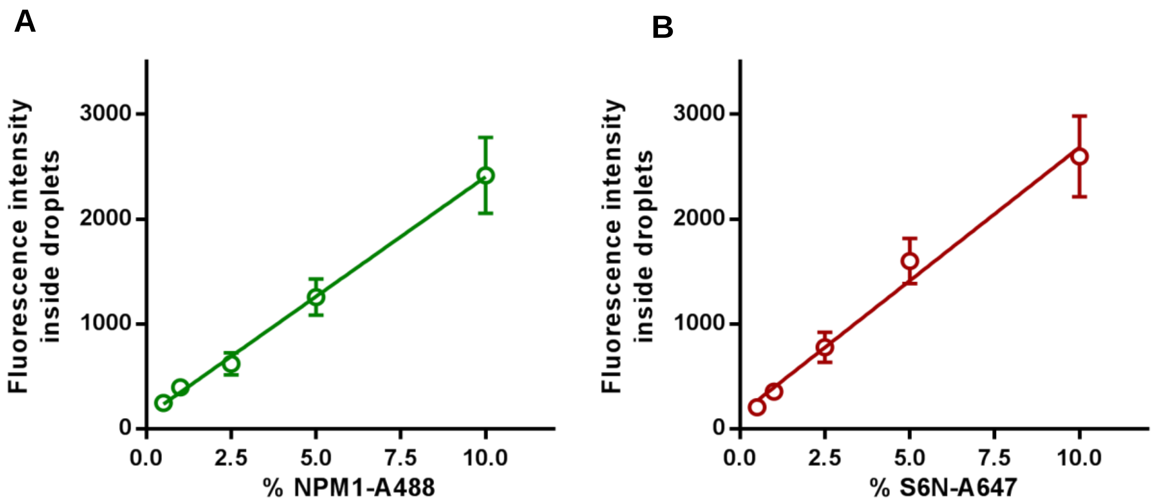

Supplementary Fig. 3. Correlation of fluorescence intensities inside droplets and concentrations of fluorescently-labeled proteins. Mean fluorescence intensities of NPM1-S6N droplets (5 μM NPM1, 5 μM S6N) in 5% PEG containing different percentages of NPM1-488 and S6N-647 were determined from confocal microscopy images. Error bars, mean ± s.d, n ≥ 40. Fluorescence intensities linearly increased with higher percent of NPM1-A488 (in A; R^2=0.9975) and S6N-647 (in B; R^2=0.9879) inside droplets.

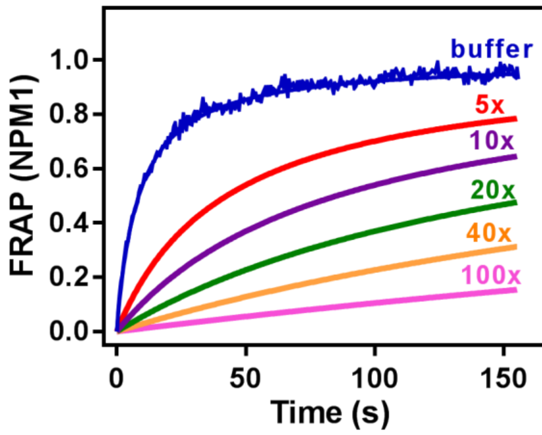

Supplementary Fig. 4. Theoretical FRAP recovery curves as a function of increase in the viscosity within droplets. Experimental FRAP recovery curve for NPM1-S6N droplets (10 μM NPM1:10 μM S6N) in buffer without crowding agent (blue trace) and theoretical recovery curves for NPM1-A488 within droplets in which the viscosity of the dense phase was increased by 5-fold (red), 10-fold (violet), 20-fold (green), 40-fold (orange) and 100-fold (pink).

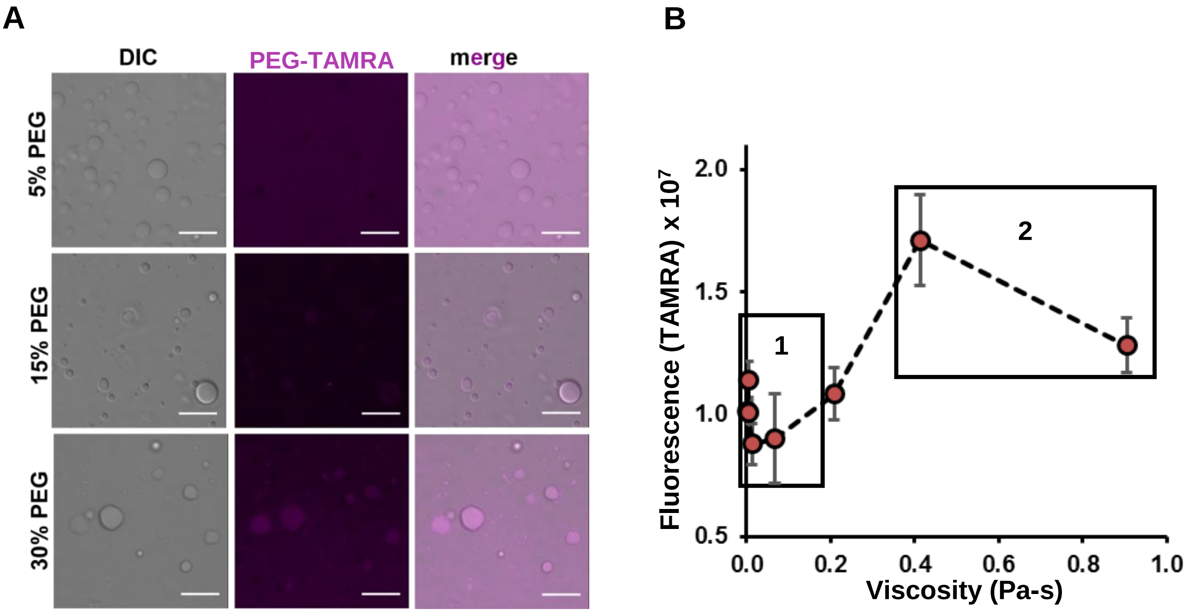

Supplementary Fig. 5. Distribution of PEG-TAMRA molecules in the dense and light phases of NPM1 homotypic droplets in buffers containing different percentages of PEG. A) Fluorescence microscopy images of 10 μM PEG-TAMRA mixed with 10 μM NPM1 droplets in the presence of 5%, 15% and 30% PEG; scale bar = 10 μm. **B)** Effect of increased viscosity on TAMRA fluorescence. Fluorescence of 5 μM free TAMRA dye in standard water-glycerol solutions with varying viscosities were determined using a plate reader. Values represent mean ± s.d. for n = 5. Correction factor of 1.5 was determined by dividing the average fluorescence in box 2 over the average fluorescence in box 1.

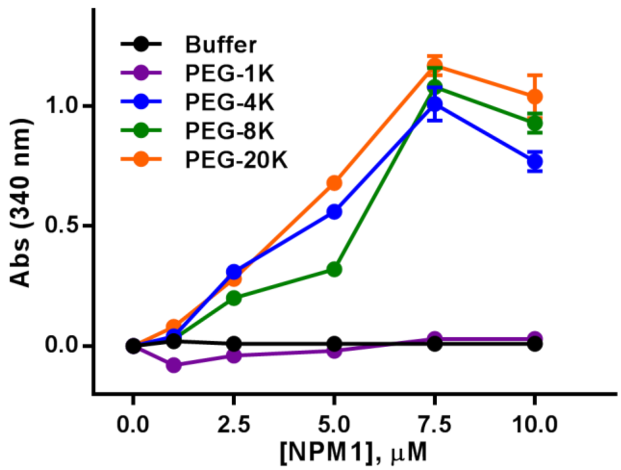

Supplementary Fig. 6. Effect of crowder size on homotypic LLPS of NPM1. Turbidity measurements for protein solutions prepared with different NPM1 concentrations in 15% PEG of different molecular weights, 1 kDa (violet circles), 4 kDa (blue circles), 8 kDa (green circles) and 20 kDa (orange circles) in 10 mM Tris, 150 mM NaCl, 2 mM DTT, pH 7.5 buffer. Shown in black circles are turbidities of NPM1 solutions in buffer only. Turbidity was determined by measuring the absorbance at 340 nm; measurements were made 10 min after mixing. Values represent mean ± s.d., n = 3.

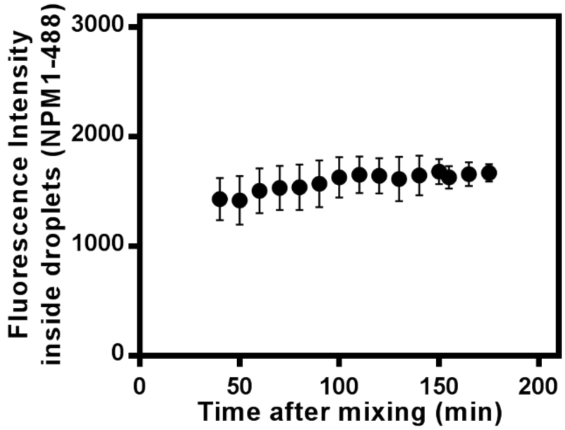

Supplementary Fig. 7. **The concentration of NPM1 within homotypic droplets in the presence of 5% PEG exhibits minimal increase during the gelation process.** Mean fluorescence intensities of NPM1-A488 from microscopy images of homotypic NPM1 droplets acquired at different time points after droplet formation. Droplets were prepared by mixing 20 μM NPM1 (5% NPM1-A488) with 5% PEG in buffer. Mean fluorescence intensities of multiple droplets were determined at different time points. Values represent mean ± s.d. for n ≥ 30 droplets.

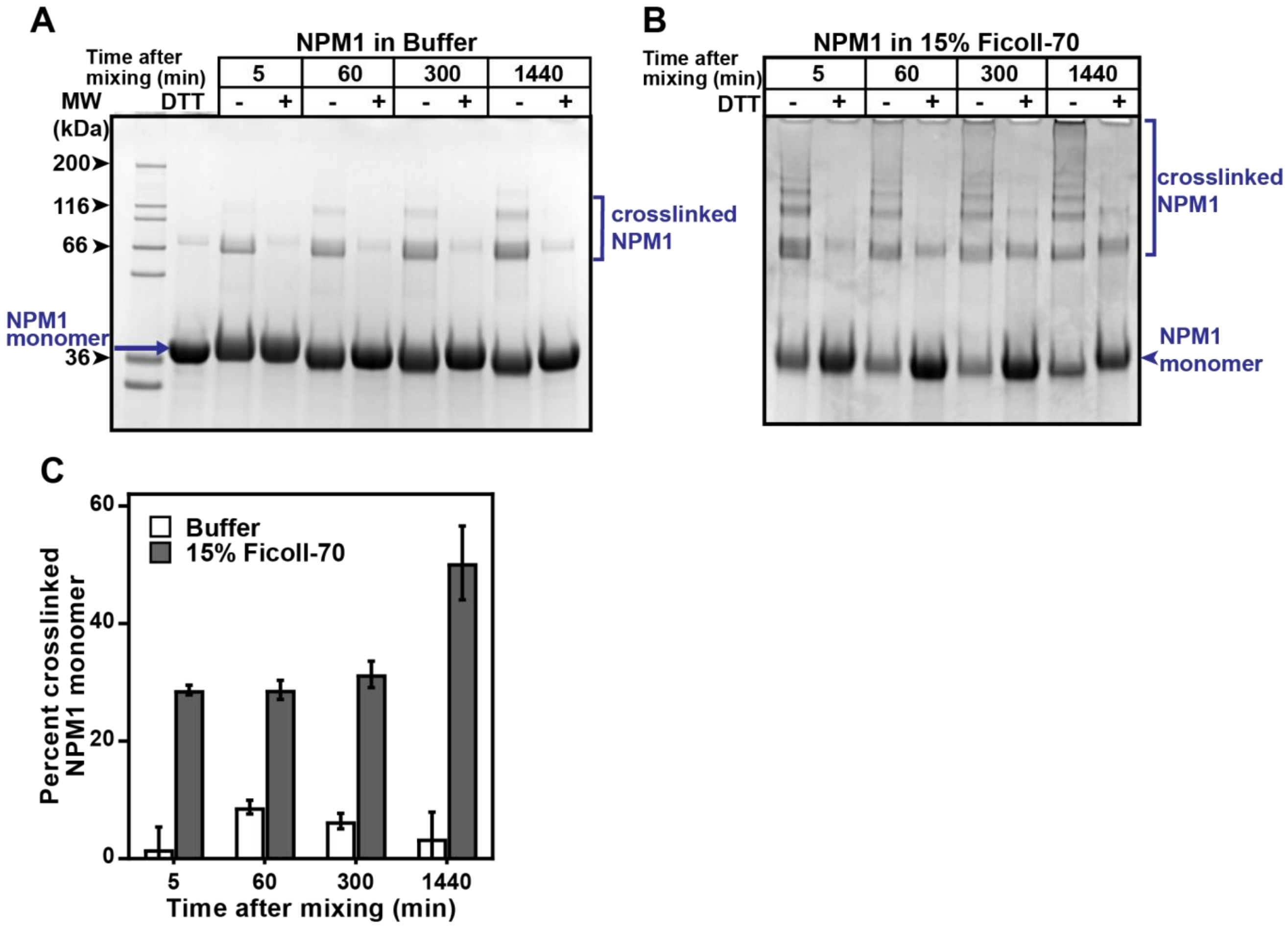

Supplementary Fig. 8. **Covalent cross-linking of NPM1 using DSP in the presence and absence of Ficoll-70 at different times after mixing.** NPM1 solutions were treated with DSP (NPM1:DSP molar ratio of 1:2.5) for two minutes. Cross-linked NPM1 solutions were analyzed using SDS-PAGE in the absence and presence of reducing agent. The percentage of cross-linked NPM1 was determined from the ratio of band intensities for monomeric NPM1 in the non-reduced and reduced NPM1 samples. Values represent mean ± s.d. for n = 3.

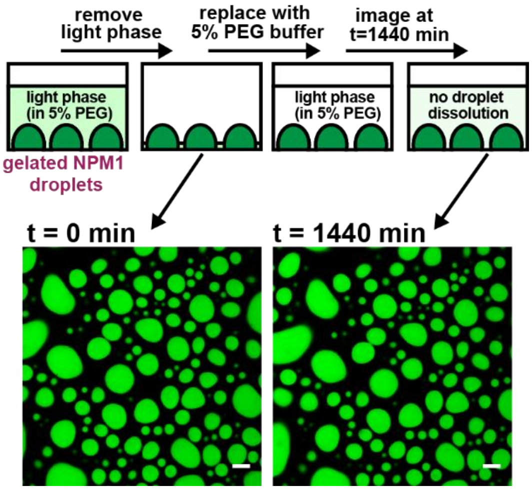

Supplementary Fig. 9. Fluorescence microscopy images of gelated NPM1 droplets exchanged with fresh 5% PEG-containing buffer. Droplets containing NPM1-A488 were incubated for 24 hours on a chambered glass slide at room temperature in 5% PEG-containing buffer. Light phase (90% of the solution) was gently removed and replaced with the same volume of 5% PEG-containing buffer. Droplets were monitored over time using confocal fluorescence microscopy imaging. An image recorded 1440 minutes after buffer exchange is shown (right) in comparison with an image recorded immediately after buffer removal. Only a small decrease in mean NPM1-A488 fluorescence intensities (11%) within droplets was observed after 24 h.

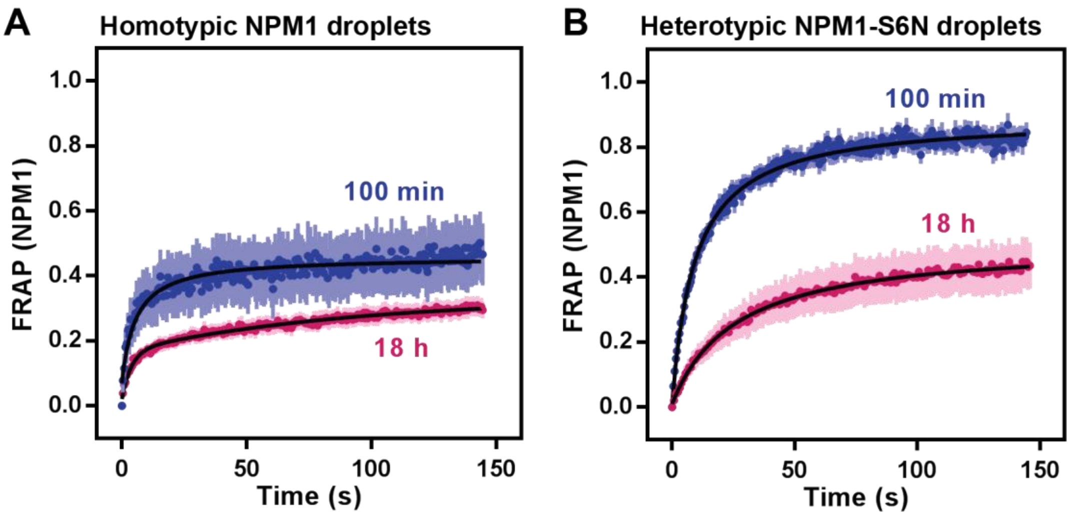

Supplementary Fig. 10. Both homotypic NPM1 and heterotypic NPM1-S6N droplets experience time-dependent gelation, as reflected by reduced NPM1 mobility within droplets. FRAP curves for recovery of NPM1-A488 within homotypic NPM1 (prepared with 20 μM NPM1) (**A**) and heterotypic NPM1-S6N droplets (prepared with 10 μM NPM1 and 10 μM S6N) (**B**) in the presence of 5% PEG, recorded 100 min (blue traces) and 18 h (red traces) after droplet preparation. Mf of NPM1-A488 within homotypic NPM1 droplets was ~45% and ~ 30% after incubating for 100 min and 18 h, respectively. The corresponding values for NPM1-A488 within the heterotypic NPM1-S6N droplets were ~90% and 50% after incubation times of 100 min and 18h, respectively. The black traces are fitted curves. Values represent mean ± s.d. for n ≥ 8 droplets.

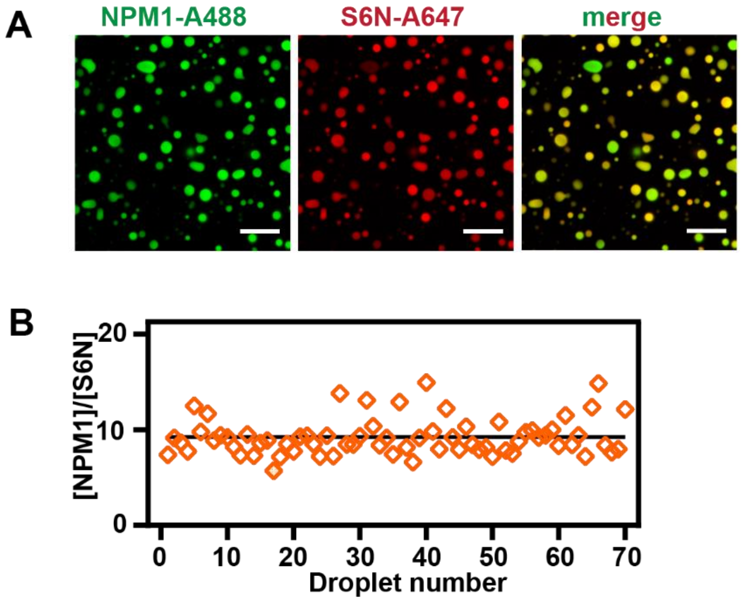

Supplementary Fig. 11. Stochastic LLPS of NPM1 with S6N pre-mixed with crowding agent. (**A**) Confocal microscopy images of NPM1-A488 (green) and S6N-A647 (red) within heterotypic NPM1-S6N droplets prepared by adding 10 μM NPM1 (in buffer) to 10 μM S6N dissolved in buffer containing PEG. The images were recorded 4 h after mixing at a final PEG concentration of 15%; scale bar =10 μm. (**B**) Ratios of concentrations of NPM1 and S6N within individual droplets. Using the order of solution mixing described in (**A**), droplets stochastically form with [NPM1]/[S6N] values that vary between ~8 and ~15.

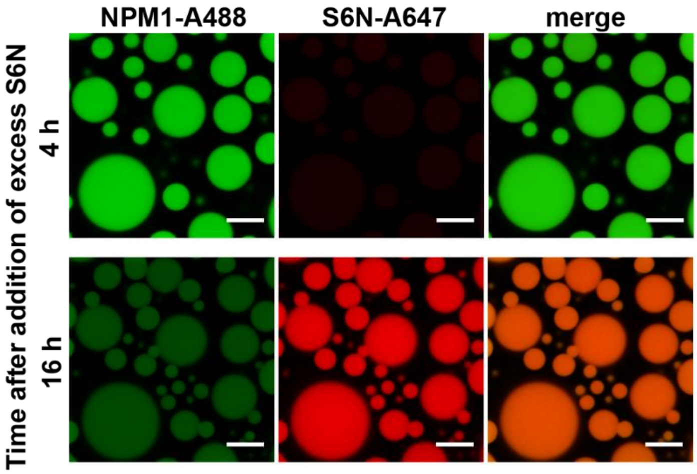

Supplementary Fig. 12. Formation of *de novo* NPM1-S6N droplets as NPM1 is expelled from the heterotypic NPM1-S6N scaffold upon addition of excess S6N. Confocal microscopy images of droplets in 5% PEG 4 hours (top) and 16 h (bottom) after S6N addition. NPM1-A488 is shown in green and S6N-A647 is in red; scale bar = 10 μm. Images show that as S6N accumulates inside the droplets and NPM1 is expelled, (see Fig. 5D) NPM1 becomes available to form new droplets over time.

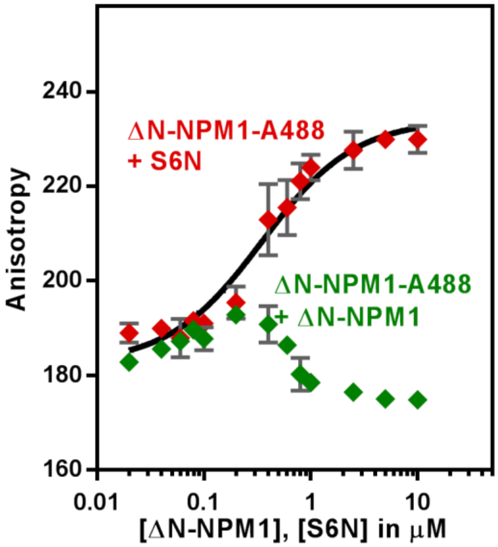

Supplementary Fig. 13. Binding of NPM-IDR to S6N and to itself monitored by fluorescence anisotropy. Binding isotherms were generated by measuring the fluorescence anisotropy of 20 nM ΔN-NPM1-A488 with increasing concentrations of S6N (red) or ΔN-NPM1 (green). The K_D for S6N:ΔN-NPM1 was determined from the fitted binding curve to be 350 ± 82 nM. The K_D for ΔN-NPM1:ΔN-NPM1 cannot be determined with this method; Values represent mean ± s.d. for n = 3.

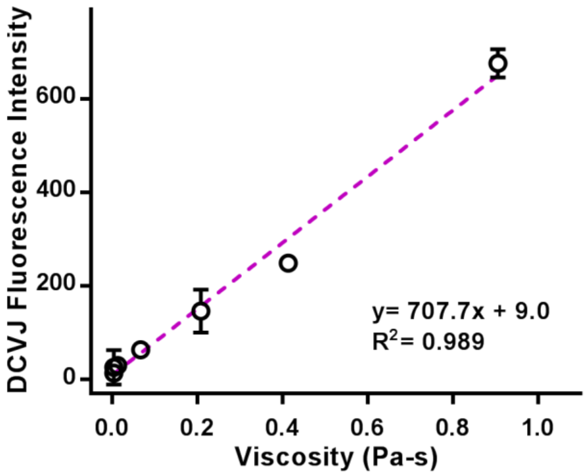

Supplementary Fig. 14. Linear correlation between viscosity of glycerol solutions and fluorescence of molecular rotor, DCVJ. Standard glycerol/water solutions with different mass fractions were prepared and mixed with 10 μM DCVJ; fluorescence intensity was measured by confocal fluorescence microscopy. Values represent mean fluorescence intensities ± s.d. across the entire image.

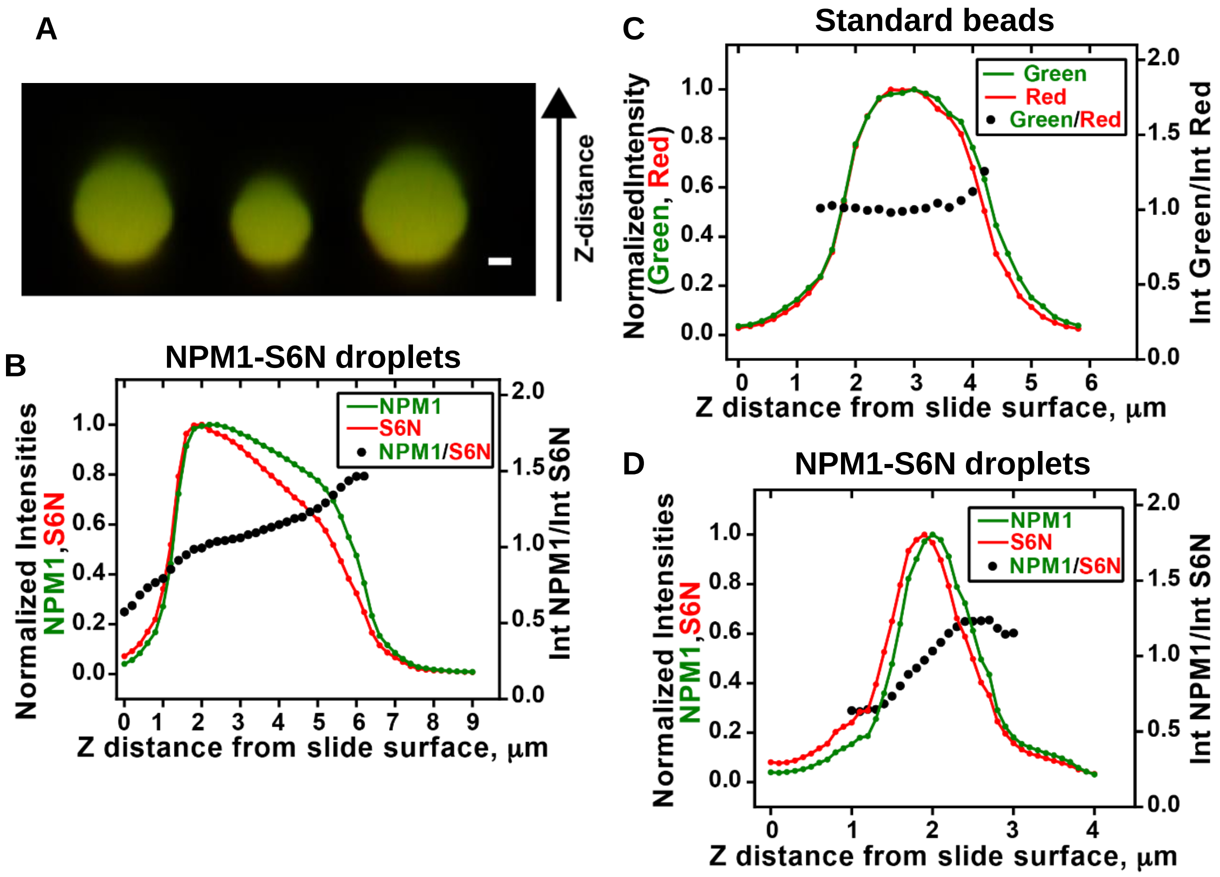

Supplementary Fig. 15. Heterogeneous composition of NPM1-S6N droplets across the Z plane. (A) Merged Z-stack reconstructions of NPM1-A488 (green) and S6N-A647 (red) channels of NPM1-S6N droplets (1:1 ratio) in 5% PEG showing heterogeneity in color distribution across the Z-plane, scale bar = 1 µm. **(B)** Plot of the Z-profile intensities of NPM1-A488 (green trace, left axis) and S6N-A647 (red trace, left axis) of the center of the droplet images on a hydrophilic surface. The black circles (right axis) are the corresponding ratios (NPM1/S6N) of the intensities across the Z-plane. **(C)** Plot of the Z-profile intensities from green (green trace, left axis) and red (red trace, left axis) channels of the center of a 4-µm TetraSpeck microsphere bead. The black circles (right axis) are the corresponding ratios (green/red) of the intensities across the Z-plane. **(D)** Plot of the Z-profile intensities of NPM1-A488 (green trace, left axis) and S6N-A647 (red trace, left axis) of the center of the droplet imaged on a hydrophobic surface. The black circles (right axis) are the corresponding ratios (NPM1/S6N) of the intensities across the Z-plane.

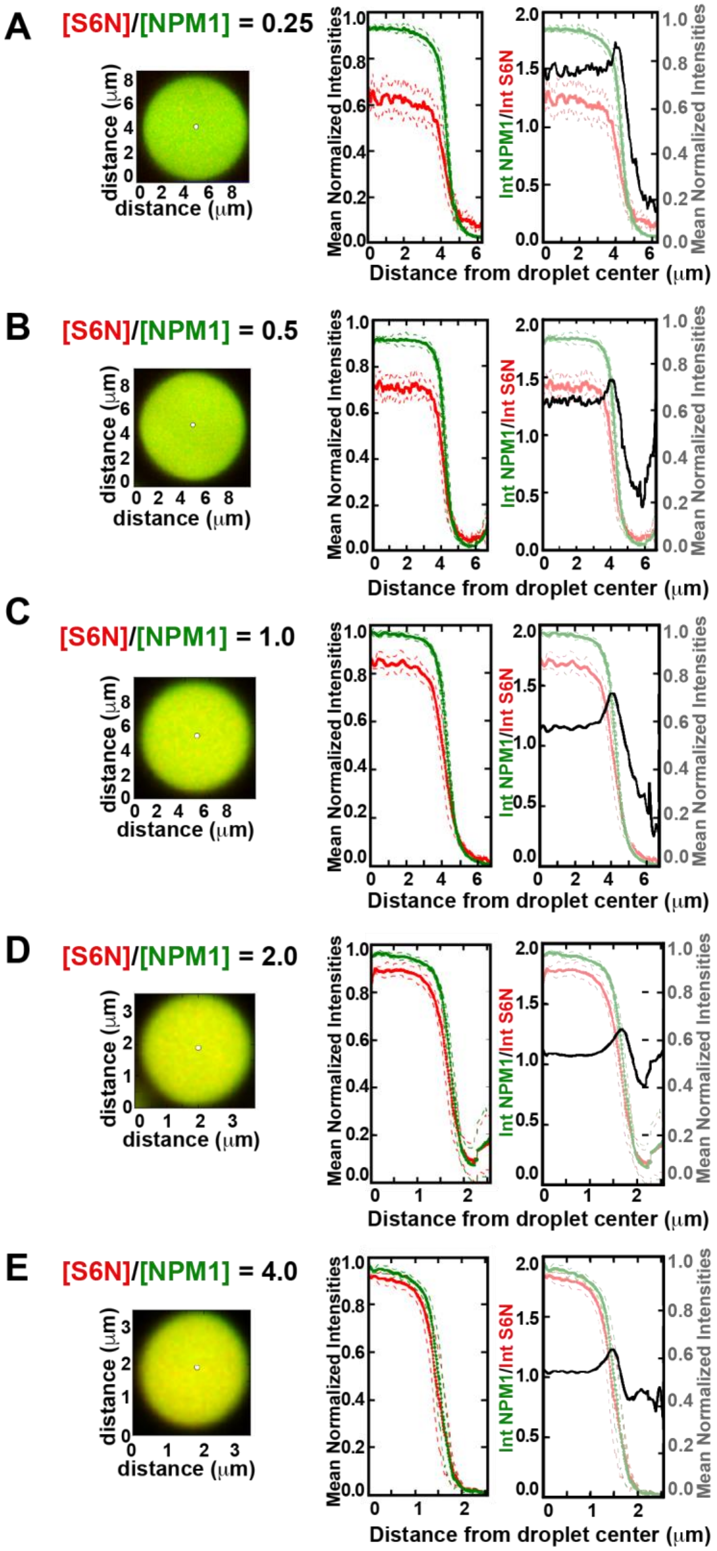

Supplementary Fig. 16. An NPM1-enriched layer is present in variable blends of NPM1-S6N heterotypic droplets. Analysis of radial fluorescence intensities of NPM1-A488 and S6N-647 using Sauron for [S6N]/[NPM1] values: (**A**) 0.25, (**B**) 0.5 (**C**) 1.0 (**D**) 2.0 and (**E**) 4.0. Left panels show overlays of fluorescence microscopy images of NPM1-A488 (green) and S6N-A647 (red) of the NPM1-S6N droplet in the presence of 5% PEG. The white circle marks the droplet center determined by Sauron. Middle panels show mean values of normalized radial intensities (solid lines) for NPM1-A488 (green) and S6N-A647 (red) for NPM1-S6N droplet plotted *versus* distance from the droplet center determined from Sauron. Standard deviations of mean intensity values for n = 30 radii are shown in dashed lines. Right panels show ratio of mean normalized intensities for NPM1-A488 and S6N-647 (left axis) plotted *versus* the distance from the droplet center. The mean intensity traces (from middle panels) are shown for reference (right axis).

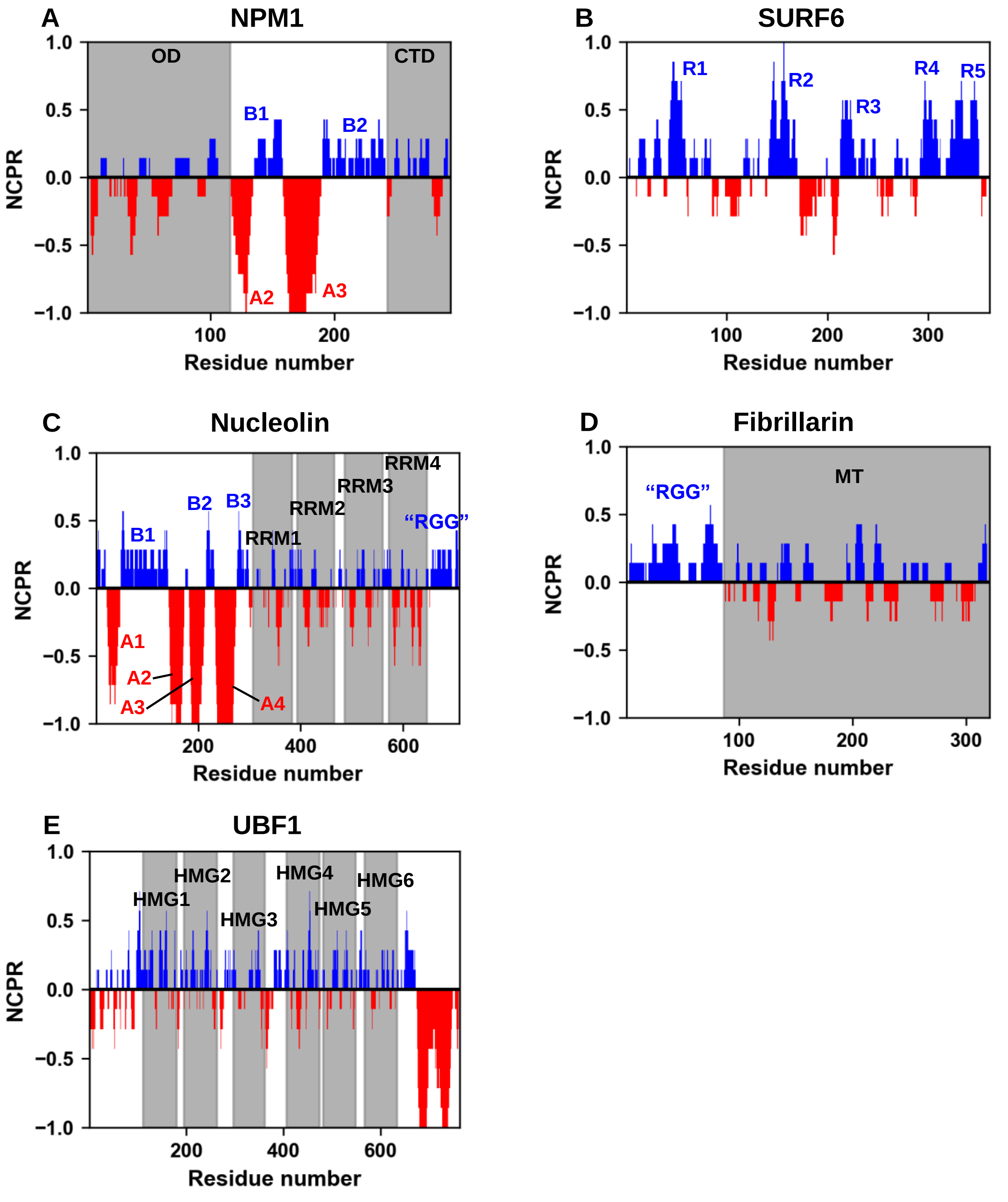

Supplementary Fig. 17. Abundant non-ribosomal nucleolar proteins display alternate charge patterning in their primary structure. Net charge per residue plots of **(A)** NPM1, **(B)** SURF6, **(C)** Nucleolin, **(D)** Fibrillarin, **(E)** UBF1, calculated with CIDER[1] (http://pappulab.wustl.edu/CIDER/analysis/). Grey shaded boxes indicate positions of folded domains; OD - oligomerization domain; CTD – C-terminal nucleic acid binding domain; RRM – RNA recognition motif; MT – methyltransferase domain; HMG – high mobility group box.

SUPPLEMENTARY REFERENCES

1. Pollard, T.D. A guide to simple and informative binding assays. *Mol Biol Cell* **21**, 4061-4067 (2010).
2. Rashid, R., Chee, S.M., Raghunath, M. & Wohland, T. Macromolecular crowding gives rise to microviscosity, anomalous diffusion and accelerated actin polymerization. *Phys Biol* **12**, 034001 (2015).
3. Holehouse, A.S., Das, R.K., Ahad, J.N., Richardson, M.O. & Pappu, R.V. CIDER: Resources to Analyze Sequence-Ensemble Relationships of Intrinsically Disordered Proteins. *Biophys J* **112**, 16-21 (2017).
